# The Changing Demography of Late-Life Family Caregiving: A Research Agenda to Understand Future Care Networks

**DOI:** 10.1093/geroni/igaf122.297

**Published:** 2025-12-31

**Authors:** I-Fen Lin

**Affiliations:** University of Missouri, Columbia, Missouri, United States

## Abstract

Repeated claims that a dwindling supply of potential caregivers is creating a crisis in care for the U.S. aging population have not been well grounded in empirical research. Concerns about the supply of family care do not adequately recognize factors that may modify the availability and willingness of family and friends to provide care to older persons in need of assistance or the increasing heterogeneity of the older population. In this paper, we set forth a framework that places family caregiving in the context of older adults’ care needs, the alternatives available to them, and the outcomes of that care. We focus on care networks, rather than individuals, and discuss the demographic and social changes that may alter the formation of care networks in the future. Last, we identify research areas to prioritize in order to better support planning efforts to care for the aging U.S. population.

